# Inhibition of the immunoproteasome LMP2 ameliorates ischemia/hypoxia-induced blood–brain barrier injury through the Wnt/β-catenin signalling pathway

**DOI:** 10.1186/s40779-021-00356-x

**Published:** 2021-12-03

**Authors:** Xing-Yong Chen, Shao-Fen Wan, Nan-Nan Yao, Ze-Jing Lin, Yan-Guang Mao, Xiao-Hua Yu, Yin-Zhou Wang

**Affiliations:** 1 Department of Neurology, Fujian Provincial Hospital, Shengli Clinical Medical College of Fujian Medical University, No. 134, Dongjie, Gulou District, Fuzhou, 350001 Fujian China; 2 Fujian Academy of Medical Science, Fuzhou, 350001 Fujian China; 3 Key Testing Laboratory of Fujian Province, Fuzhou, 350001 Fujian China

**Keywords:** Immunoproteasome, Blood–brain barrier, Wnt/β-catenin pathway, Oxygen–glucose deprivation/reperfusion, Cerebral ischemia

## Abstract

**Background:**

Disruption of the blood–brain barrier (BBB) after a stroke can lead to brain injury and neurological impairment. Previous work confirmed the involvement of the immunoproteasome subunit of low molecular mass peptide 2 (LMP2) in the pathophysiology of ischemia stroke. However, the relationship between the immunoproteasome LMP2 and the BBB remains unclear.

**Methods:**

Adult male Sprague–Dawley rats were subjected to transient middle cerebral artery occlusion/reperfusion (MCAO/R). Three days before MCAO, the rats were treated with lentivirus-mediated LMP2 shRNA preparations by stereotactical injection into the ipsilateral hemispheric region. The rat brain microvascular endothelial cell (RBMVEC) line was exposed to oxygen–glucose deprivation/reperfusion (OGD/R) to mimic ischemic conditions in vitro. The RNA interference-mediated knockdown of LMP2 or β-catenin was analysed in vivo and in vitro. Analysis of the quantity of extravasated Evans blue (EB) and cerebral fluorescent angiography were performed to evaluate the integrity of the BBB. Immunofluorescence and Western blotting were employed to detect the expression of target proteins. Cell migration was evaluated using a scratch migration assay. The results of immunofluorescence, Western blotting and cell migration were quantified using the software ImageJ (Version 1.53m). Parametric data from different groups were compared using one-way ANOVA followed by the least significant difference (LSD) test.

**Results:**

Cerebral ischemia led to lower levels of structural components of the BBB such as tight junction proteins (occludin, claudin-1 and ZO-1) in the MCAO/R group compared with the sham group (*P* < 0.001). However, inhibition of the immunoproteasome LMP2 restored the expression of these proteins, resulting in higher levels of occludin, claudin-1 and ZO-1 in the LMP2-shRNA group compared with the control-shRNA group (*P* < 0.001). In addition, inhibition of the immunoproteasome LMP2 contributed to higher microvascular density and decreased BBB permeability [e.g., the quantity of extravasated EB: LMP2-shRNA group (58.54 ± 7.37) µg/g *vs.* control-shRNA group (103.74 ± 4.32) µg/g, *P* < 0.001], and promoted the upregulation of Wnt-3a and β-catenin proteins in rats following MCAO/R. In vitro experiments, OGD/R induced marked upregulation of LMP2, proapoptotic protein Bax and cleaved caspase-3, and downregulation of occludin, claudin-1, ZO-1 and Bcl-2, as well as inhibition of the Wnt/β-catenin pathway Wnt-3a and β-catenin proteins in RBMVECs, compared with the control group under normal culture conditions (*P* < 0.001). However, silencing of LMP2 gene expression reversed these protein changes and promoted proliferation and migration of RBMVECs following OGD/R. Silencing of β-catenin by transfection of RBMVECs with β-catenin-siRNA aggravated the downregulation of tight junction proteins, and reduced the proliferation and migration of RBMVECs following OGD/R, compared with the control-siRNA group (*P* < 0.001). LMP2-siRNA and β-catenin-siRNA co-transfection partly counteracted the beneficial effects of silencing LMP2-siRNA on the levels of tight junction proteins in RBMVECs exposed to OGD/R.

**Conclusion:**

This study suggests that inhibition of the immunoproteasome LMP2 ameliorates ischemia/hypoxia-induced BBB injury, and that the molecular mechanism involves the immunoproteasome-regulated activation of the Wnt/β-catenin signalling pathway under ischemic conditions.

**Supplementary Information:**

The online version contains supplementary material available at 10.1186/s40779-021-00356-x.

## Background

It is universally acknowledged that stroke is a primary cause of morbidity, disability and mortality. Ischemic stroke is the most prevalent form of stroke worldwide. A large number of factors, including inflammatory reactions and oxidative stress, are involved in the progression of cerebral ischemia injury. Among these, disruption of the blood–brain barrier (BBB) facilitates injury progression, increases the risk of haemorrhage and is predictive of a poor outcome [1, 2]. The structural components of the BBB include endothelial cells and their linking tight junctions, pericytes, astrocytic endfeet and extracellular matrix components, which are collectively referred to as the neurovascular unit. BBB tight junction formation primarily involves specific transmembrane proteins (i.e., claudins, occludin, tricellulin and junctional adhesion molecules) that are linked to cytoskeletal filaments by interactions with accessory proteins [i.e., zonula occludens (ZO) protein]. Transmembrane tight junction proteins are vital for the maintenance of functional BBB integrity [3]. For example, claudins contribute to the physiological seal of the tight junction. Barrier function can be influenced by claudin-1, claudin-3, claudin-5 and claudin-12. Occludin is a critical transmembrane regulator of BBB functional integrity in vivo. ZO-1, a membrane-associated guanylate kinase-like protein, links transmembrane tight junction proteins to the actin cytoskeleton. Dissociation of ZO-1 from the junction complex causes increased BBB permeability [3]. BBB destruction is associated with abnormal expression of these tight junction proteins and BBB transporters [4]. BBB controls the homeostasis of the central nervous system (CNS) and protects brain tissue from exposure to potentially toxic substances. Therefore, BBB disruption after ischemic stroke leads to severe pathological consequences and exacerbates brain injury. Although the complex mechanism of BBB injury after stroke is still not fully understood, exploration of new intrinsic or exogenous methods and potential therapeutic agents aimed at ameliorating ischemia/reperfusion injury has attracted increasing attention and become an area of intensive research. In particular, antioxidant nanomaterials [5] and proteasome inhibitors [6] are expected to be effective candidate treatments for ischemia stroke.

Proteasome inhibition, which has been reported in animal models of ischemic stroke, is a promising strategy for the treatment of stroke [6, 7]. Treatment with proteasome inhibitors effectively reduces the infarct volume and neuroinflammation, enhances angioneurogenesis and stabilizes the BBB integrity [6–8]. However, the limited clinical application of general proteasome inhibitors is due to their nonselective inhibition of protein degradation. Thus, selectively targeting proteasome elements would improve efficacy and reduce side effects. Recently, research has focused on the role of the immunoproteasome in many fields [9–11]. The immunoproteasome is a subtype of proteasome that is predominantly present in immune cells. Structurally, the immunoproteasome contains three major catalytic subunits: LMP2 (low molecular weight protein 2, PSMB9, β1i), LMP5 and LMP7. Dysregulation of the immunoproteasome has been linked with a variety of diseases such as autoimmune diseases [12], cerebral ischemia [9, 10] and atherosclerosis [11]. Our previous work found that the immunoproteasome is involved in the pathophysiology of ischemia stroke, while inhibition of LMP2 suppresses proinflammatory cytokine production, reduces infarction volumes and enhances angiogenesis in a rat ischemia stroke model [9, 10]. Clinically, we found that elevated plasma immunoproteasome levels helped predict early haemorrhagic transformation and poor prognosis in acute ischemic stroke patients [13, 14]. BBB disruption after stroke is regulated by the actions of different factors including proteasomes, inflammatory modulators and oxidative pathways, which usually work in concert with each other at different stages of cerebral ischemia [1, 15]. Indeed, BBB injury is an early pathological event in ischemic stroke that occurs prior to the onset of neuronal injury. However, whether the immunoproteasome regulates the expression of BBB structure proteins and BBB integrity has not yet been fully elucidated. Therefore, the aim of this study was to explore the relationship between immunoproteasome LMP2 and the BBB and the molecular mechanism involved.

## Methods

### Ethical approval and experimental animals

All experiments were approved by the Institutional Animal Ethical Committee of Fujian Medical University (No. FJMUIACUC2020-0059) and performed according to the guidelines of the US Department of Health for the Use and Care of Laboratory Animals. Adult male Sprague–Dawley rats (weight 230–250 g) were included in the study. Rats were randomly assigned into three groups (each group *n* = 8): sham group, LMP2-shRNA group [rats were injected with lentivirus-mediated LMP2 short hairpin RNA (shRNA)] and control-shRNA group (rats were injected with control lentivirus vector carrying scrambled shRNA).

### Middle cerebral artery occlusion (MCAO) model

Rats were anesthetized and subjected to MCAO as described previously, with minor modifications [9]. In brief, a midline neck incision was made, and the right common carotid artery, external carotid artery and internal carotid artery were isolated. The external carotid artery was tied. A 4–0 monofilament nylon suture (Beijing Sunbio Biotech Co. Ltd., Beijing, China) with a rounded tip was aseptically inserted from the right common carotid artery to the internal carotid artery through the stump of the external carotid artery and gently advanced to occlude the middle cerebral artery. Recirculation/reperfusion of the cerebral blood flow was allowed by gently removing the monofilament after one hour of ischemia, followed by 14 days of reperfusion. In sham-operated animals, all procedures except occlusion of the MCA were performed.

### Lentiviral construction preparation and injection

According to our previous study [9], four shRNA sequences targeting rat LMP2 (GenBank, Psmb9, NM_012708) and a negative control sequence were constructed by Genechem (Shanghai, China). Lentivirus-mediated LMP2 shRNA preparations were constructed and infused stereotactically into the ipsilateral hemispheric region 3 days before MCAO. Briefly, rats were anesthetized as above and placed on a stereotactic apparatus. A total volume of 10 µl of lentivirus suspension was delivered into the right ischemia region using a 15 µl syringe at the following coordinates: bregma backward 1 mm, 1.5 mm lateral, 4 mm dorsoventral.

### Evaluation of BBB disruption

The integrity of the BBB was measured using Evans blue (EB) solution (Solarbio, Beijing, China). Before being euthanized, the rats were injected via the tail vein with 2% EB (4 ml/kg). After 2 h circulation, the rats were transcardially perfused with 0.9% NaCl until the outflow fluid from the right atrium was clear. Then, the injured hemisphere was dissected, weighed and incubated in formamide solution in a 37 °C water bath. After 48 h, the supernatant was obtained by centrifugation of the tissue at 1000 × *g* for 15 min. Finally, the quantity of extravasated EB in the sample was detected by a spectrophotometer at a wavelength of 632.

### Cerebral fluorescent angiography

Cerebral fluorescent angiography was performed as described in detail elsewhere. Briefly, FITC-dextran (150 kD, 0.1 ml 50 mg/ml in double-distilled water; Sigma, USA) was administered intravenously via the rat tail vein. After 60 min, rats were euthanized. Brains were rapidly removed and placed in 4% paraformaldehyde (PFA) in 0.01 mol/L phosphate-buffered saline (PBS) at 4 °C for 24 h and then incubated in 30% sucrose in PBS for another 48 h at 4 °C. Sequential coronal Sects. (40-µm thick) were cut and observed under a fluorescence microscope.

### Cell culture

Rat brain microvascular endothelial cells (RBMVECs) involved in this study were purchased from the cell bank of Shanghai Zishi Biotechnology Co., Ltd. (Shanghai, China). Normal RBMVECs were cultured in Dulbecco’s modified Eagle’s medium (DMEM)/high glucose medium (Solarbio) containing 10% fetal bovine serum (FBS, AusGeneX, Australia) and 1% penicillin–streptomycin solution (Solarbio), and maintained in an incubator at 37 °C with a humidified atmosphere containing 5% CO_2_. Medium was exchanged every 3–4 days. RBMVECs were identified by immunofluorescence of vascular von Willebrand factor (VWF).

### Oxygen–glucose deprivation and reoxygenation (OGD/R)

The OGD/R model using RBMVECs was performed to mimic ischemic/hypoxic/reperfusion conditions in vitro as described previously with minor modifications [16]. Briefly, cells were washed with PBS (pH 7.4) twice and cultured in glucose-free DMEM (Solarbio) without FBS. Then, cells were placed into a hypoxic incubator chamber (Changjing Biotech Co. Ltd., Changsha, China) containing a gas mixture composed of 5% CO_2_ and 95% N_2_ at 37 °C for 1 h of hypoxia followed by a return to normoxic conditions with glucose-containing DMEM supplemented with 10% FBS for 24 h reoxygenation in a humidified atmosphere containing 5% CO_2_ at 37 °C. Control cells were cultured in DMEM and treated similarly to those of the experimental groups.

### siRNA transfection in RBMVECs

RBMVECs were seeded into 6-well plates in 2 ml of antibiotic-free DMEM supplemented with FBS and then maintained at 37 °C for 24 h until they reached 70–80% confluence. During transfection, Lipofectamine® RNAiMAX (Invitrogen, Carlsbad, CA, USA) was used according to the manufacturer’s instructions. The siRNA duplexes targeting LMP2 (LMP2-siRNA) or β-catenin (β-catenin-siRNA) were purchased from RiboBio, Co., Ltd. (Guangzhou, China) and their sequences were as follows: LMP2-siRNA: Si1 (TGAAGAACATCTCCTACAA), Si2 (TAGTGAACCGCGTGTTTGA), Si3 (GCACCTATATTTACGGTTA); β-catenin-siRNA: Si1 (ACATCGAAGACTCTACAAT), Si2 (TAGTGATTGAACCGCGTGT), Si3 (ACCGCGTGTAGACTCAATG). After 48 h transfection, cells were used in subsequent experiments.

### Scratch migration assay

The scratch or wound healing assay, which involves measuring cell migration across a gap induced by a scratch injury to the monolayer of cells, is the method of choice for studying cell migration due to its simplicity and low cost. Cells were seeded into 6-well plates, and the monolayer was then gently scratched with a sterile 200-µl pipette tip. Cell migration into the gap was monitored at 0, 24 and 48 h by phase-contrast microscopy. Cell migration was evaluated by a widely used quantification method, which we term the area method, as previously described [17]. Briefly, to assess migration in an indirect manner, the wound healing (WH) percentage was tracked: WH = [A(t) − A(0)]/A(0) × 100%, where A(t) is the wound area at time t and A(0) is its initial area. The area was quantified using the software ImageJ (Version 1.53 m) and followed the literature [18].

### Immunofluorescence

Briefly, cells grown on coverslips in 12-well plates were fixed with freshly prepared 4% paraformaldehyde in 0.01 mol/L PBS for 30 min. After washing with PBS, the cells were blocked with 10% normal goat serum (Solarbio) for 1 h at room temperature. Then, cells were incubated with the following primary and secondary antibodies: rabbit anti-von Willebrand factor (1:400, Abcam, Cambridge, MA, USA), rabbit anti-CD31 (1:200, Abcam), mouse anti-LMP2, mouse anti-occludin and mouse anti-claudin-1 (1:100, Santa Cruz Biotechnology, USA), rabbit anti-β-catenin (1:200, Cell Signaling Technology, Danvers, MA, USA), rabbit anti-ZO-1 (1:100, Invitrogen), Alexa Fluor® 594 conjugated goat anti-rabbit IgG or Alexa Fluor® 488 conjugated goat anti-mouse IgG (1:1000, Cell Signaling Technology). Finally, slides were mounted in antifade mountant with DAPI antifade reagent (Invitrogen) prior to imaging. The mean immunofluorescent intensity of each target protein was calculated using the software ImageJ (Version 1.53 m) and followed the literature [18].

### RNA extraction and real-time fluorescence quantitative PCR

In brief, after washing with 0.01 PBS (pH 7.4), the cells were lysed by TRIzol for 15 min and then transferred to a fresh 1.5-ml centrifugal tube. After the addition of 200 µl of chloroform, the tube was shaken for 1 min, and then centrifuged for 15 min (14,000×*g*). The supernatant was collected and mixed with an equal volume of isopropanol in an RNase-free centrifuge tube and the samples were centrifuged for 10 min (14,000×*g*). The supernatant was collected and mixed with 75% cold ethanol. Finally, RNA sediments were collected after centrifuging for 5 min (14,000×*g*), and were diluted using RNase-free H_2_O. Then, RNAs were reverse-transcribed into cDNAs using the PrimeScript RT kit (Takara, Dalian, China), according to the reference instructions. Gene expression was detected by reverse transcription-polymerase chain reaction (RT-PCR) assays in the ABI 7500 Fast Real-Time PCR System (Applied Biosystems, CA, USA). RNAs were quantified by the 2^−*ΔΔCT*^ method. The primer sequences used were as follows: LMP2 upstream: CATCTACTGTGCCCTCTCGG, LMP2 downstream: CAGCTACCATGAGATGCGCT; β-actin upstream: CGCGAGTACAACCTTCTTGC, β-actin downstream: CCTTCTGACCCATACCCACC.

### Western blotting analyses

Western blotting was performed as described previously [9]. Briefly, total cellular protein (20–30 µg) was separated by 12% gradient sodium dodecyl sulphate/polyacrylamide gel electrophoresis (SDS/PAGE) and then transferred onto polyvinylidene fluoride (PVDF) membrane (Millipore, USA). Membranes were blocked with Tris-buffered saline containing 0.1% Tween-20 (TBST) and 5% nonfat milk (Solarbio). The membranes were then incubated with the following primary antibodies: mouse anti-LMP2, mouse anti-occludin, mouse anti-claudin-1, mouse anti-caspase-3, mouse anti-Bax (1:500, Santa Cruz Biotechnology), rabbit anti-ZO-1 (1:1000, Invitrogen), rabbit anti-Bcl2 (1:1000, Abcam), rabbit anti-Wnt-3a (1:1000, Abcam), rabbit anti-β-catenin (1:1000, Cell Signaling Technology) and mouse anti-β-actin (1:3000, Cell Signaling Technology). The next day, horseradish peroxidase (HRP)-conjugated secondary antibodies, goat anti-mouse IgG secondary antibody (1:3000, Santa Cruz Biotechnology) and goat anti-rabbit IgG secondary antibody (1:3000, Cell Signaling Technology), were incubated with the membranes for 1 h at room temperature. Finally, immunoreactivity was detected with SuperSignal West Pico Chemiluminescent Substrate (Thermo Fisher Scientific, MA, USA) in the ChemiDoc MP Gel Imaging System (Bio-Rad, USA). The optical densities were normalized to those of β-actin and calculated as target protein expression/β-actin expression ratios [using ImageJ (Version 1.53m)]. If necessary, the blots were stripped with Restore™ Western Blot Stripping Buffer (Thermo Fisher Scientific, MA, USA) according to manufacturer’s instructions, and then the western blot procedure was repeated as described above.

### Statistical analysis

Data were analysed with software SPSS (version 20, IBM Corp., Armonk, NY, USA) and expressed as the mean ± standard deviation (SD). Parametric data from different groups were compared using one-way ANOVA followed by the least significant difference (LSD) test. *P* < 0.05 was considered statistically significant.

## Results

### LMP2 inhibition increased the levels of tight junction proteins, improved BBB integrity and upregulated Wnt/β-catenin signalling after MCAO

**Fig. 1:**
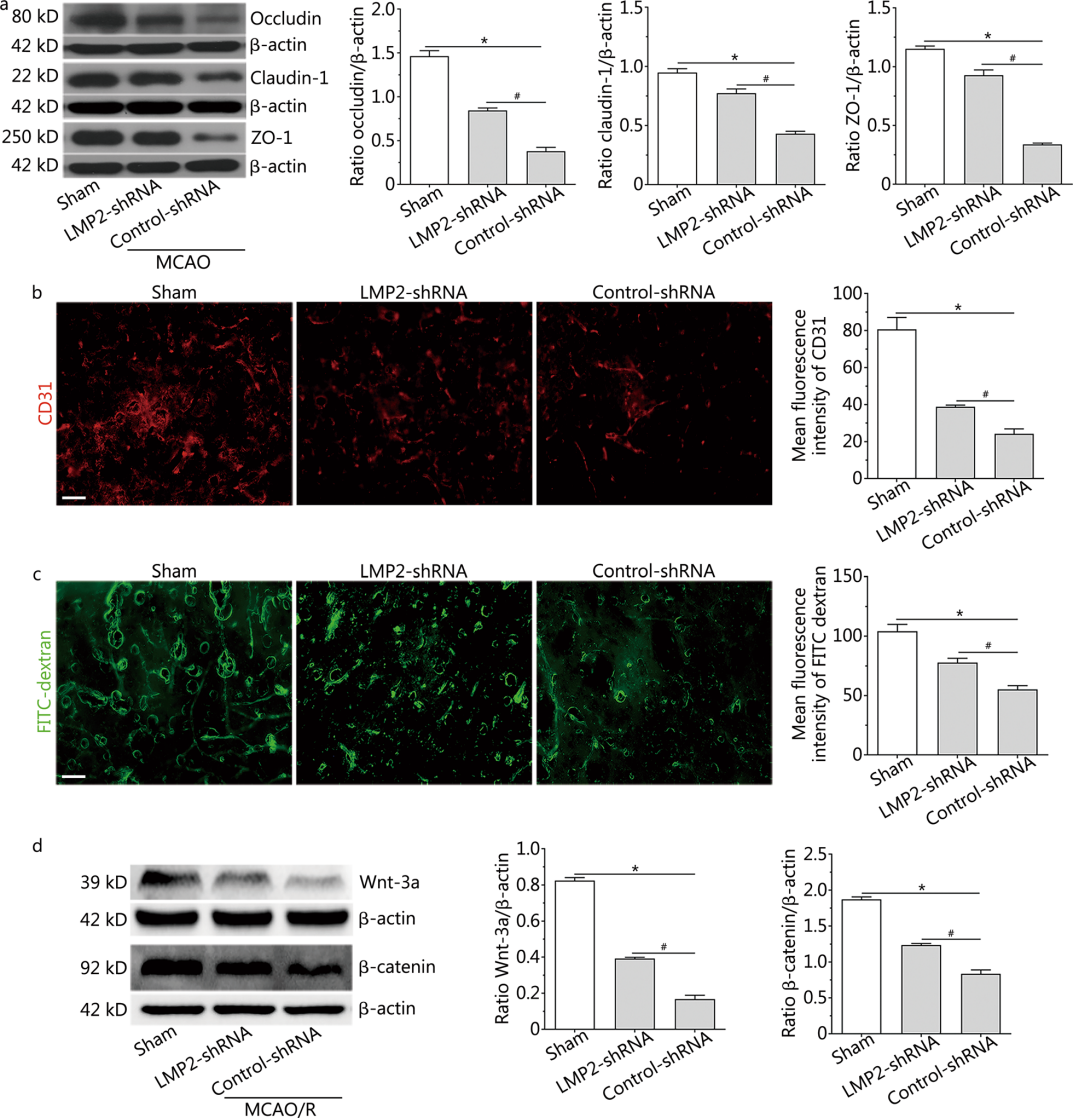
LMP2 inhibition increased the levels of tight junction proteins, improved BBB integrity and upregulated Wnt/β-catenin signalling after MCAO. **a** Western blotting showed the levels of tight junction proteins occludin, claudin-1, ZO-1 proteins in each group rats. **b** Immunofluorescence staining and quantitative analysis of CD31 in brain cortex tissue from each group rats. **c** FITC-dextran angiographic micrographs and quantitative analysis of FITC in brain cortex tissue from each group rats. **d** Representative Western blotting images of Wnt-3a and β-catenin proteins in brain cortex tissue from each group rats. Data are presented as the mean ± standard deviation (SD) from three independent experiments (each group rats *n* = 4). *P < 0.001, compared with the sham group; ^#^*P* < 0.001, compared with the control-shRNA group. All scale bars = 50 µm

Cerebral ischemia induced the elevated expression of LMP2. As shown in Additional file 1: Fig. S1, some LMP2-positive cells colocalized with CD31-positive vascular endothelial cells. To investigate the effects of LMP2 inhibition on the structural components and integrity of the BBB, we analysed tight junction proteins, such as occludin, claudin-1 and ZO-1, by Western blotting. The levels of occludin, claudin-1 and ZO-1 proteins, both in the LMP2-shRNA group and the control-shRNA group, were significantly decreased compared with the sham group (*P* < 0.001). However, inhibition of immunoproteasome LMP2 restored the expression of these proteins, with higher levels of occludin, claudin-1 and ZO-1 in the LMP2-shRNA group compared with the control-shRNA group (*P* < 0.001) (Fig. 1a).

LMP2 inhibition led to angiogenesis in rats subjected to focal cerebral ischemia/reperfusion. Immunoreactivity to vascular endothelial cell marker CD31 was significantly increased in the cortex of the peri-infarction area in rats of the LMP2-shRNA group after 14 days of ischemia/reperfusion compared with the control-shRNA group (*P* < 0.001) (Fig. 1b). Furthermore, FITC-dextran angiographic micrographs indicated high microvascular density and almost no leakage of fluorescent dyes in the sham group. By contrast, MCAO caused decreased microvascular density and increased vascular permeability, as indicated by the leakage of fluorescent dyes. Interestingly, there was higher microvascular density and decreased vascular permeability in the LMP2-shRNA group than in the control-shRNA group (*P* < 0.001) (Fig. 1c). In addition, EB exudation was increased in the MCAO/R group compared with the sham group [the quantity of extravasated EB: (15.03 ± 3.31) µg/g], but decreased in the LMP2-shRNA group compared with the control-shRNA group [the quantity of extravasated EB: LMP2-shRNA group (58.54 ± 7.37) µg/g versus control-shRNA group (103.74 ± 4.32) µg/g, *P* < 0.001].

Wnt/β-catenin signalling is necessary for maintenance and development of the structure and function of the BBB. In the present study, cerebral ischemia resulted in downregulation of the Wnt-3a and β-catenin proteins, while LMP2 inhibition rescued Wnt-3a and β-catenin protein expression in the LMP2-shRNA group compared with the control-shRNA group (*P* < 0.001) (Fig. 1d). These data suggest that inhibition of LMP2 enhances the abundance of BBB structural proteins, activates the Wnt/β-catenin signal pathway and improves BBB integrity.

### Expression of LMP2 protein in RBMVECs exposed to OGD/R

**Fig. 2:**
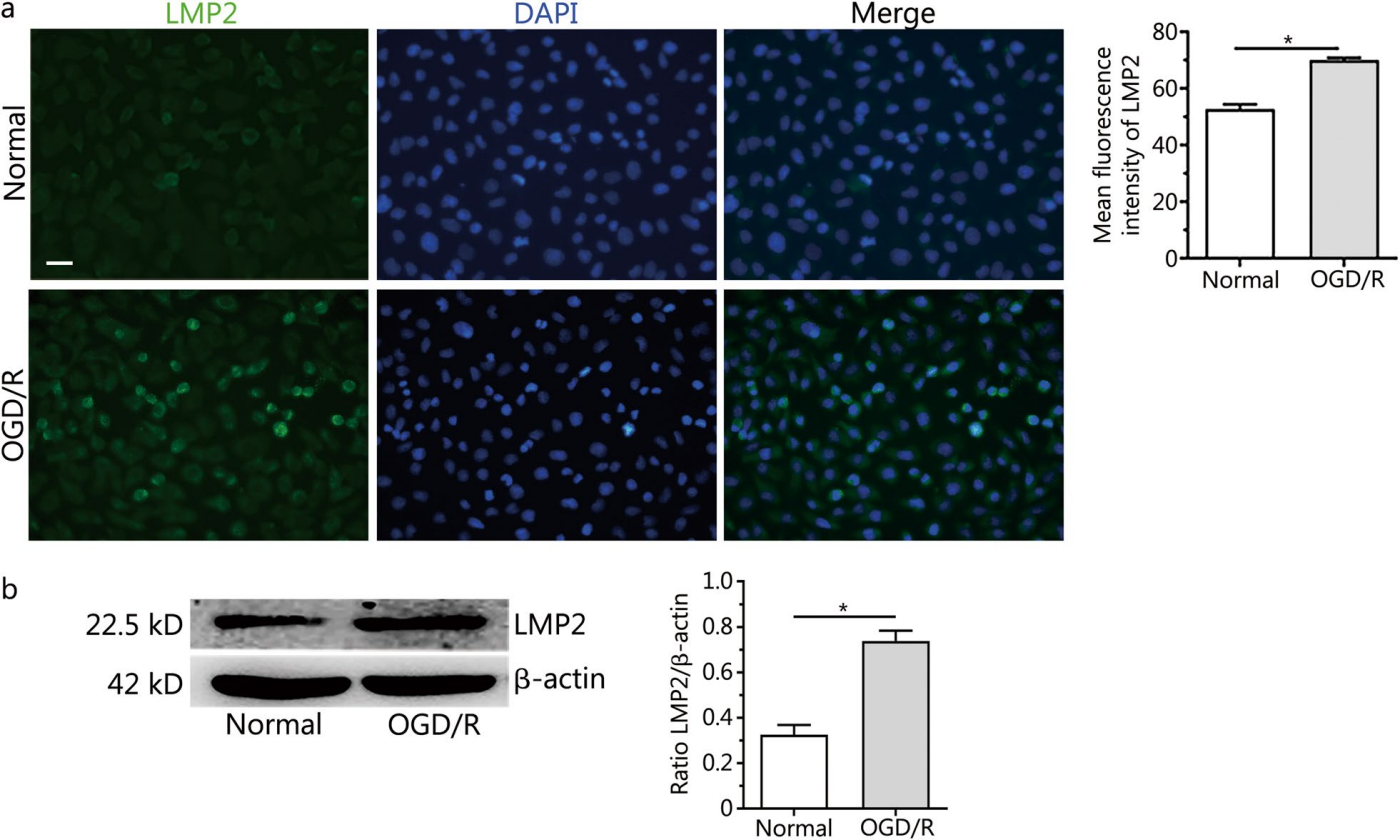
Expression of LMP2 protein in RBMVECs exposed to OGD/R. **a** Immunofluorescence staining and quantitative analysis of LMP2 in RBMVECs exposed to OGD/R. **b** Western blotting showed that the expression of LMP2 protein in RBMVECs in each group. Data are presented as the mean ± standard deviation (SD) from three independent experiments. *P < 0.001, compared with the normal group. All scale bars = 50 µm. *OGD/R* oxygen–glucose deprivation and reoxygenation. *RBMVECs* rat brain microvascular endothelial cells

The immunofluorescence of vascular VWF was observed in RBMVECs and more than 95% of cultured cells were VWF positive (Additional file 2: Fig. S2). Weak immunoreactivity of LMP2 was observed in cells under normal culture conditions. By contrast, LMP2 immunoreactivity was significantly higher in RBMVECs subjected to 1 h of OGD followed by 24 h of reoxygenation (OGD 1 h/R 24 h). LMP2 was evident in both the nucleus and cytoplasm, as indicated by co-staining with DAPI (Fig. 2a). Western blotting also showed that the level of LMP2 protein in the OGD/R group was significantly upregulated compared with the normal group (*P* < 0.001) (Fig. 2b). This suggests that OGD/R enhances the expression of LMP2 protein in RBMVECs.

### Changes to occludin, claudin-1 and ZO-1 proteins in RBMVECs after OGD/R

**Fig. 3:**
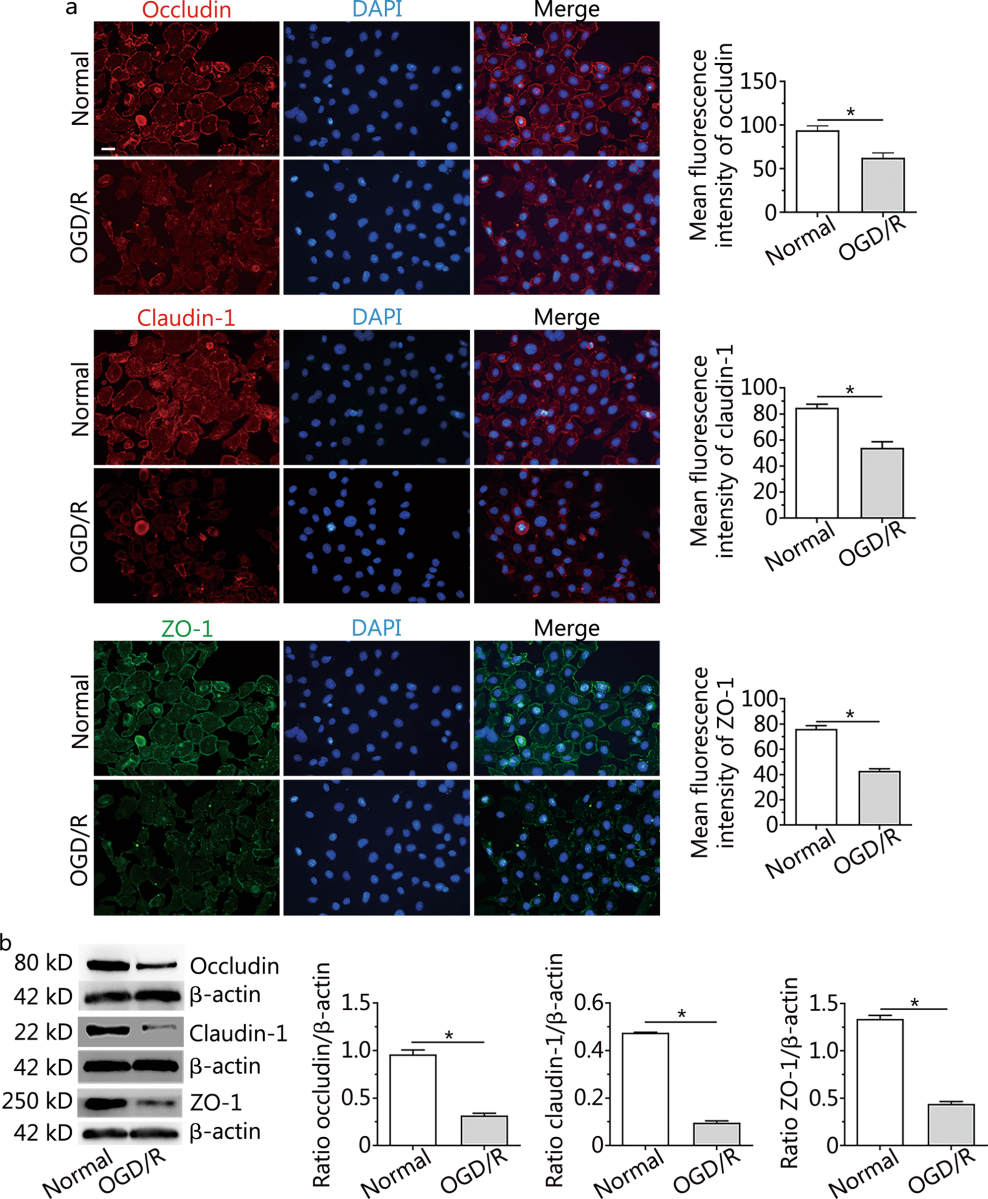
Changes to occludin, claudin-1 and ZO-1 proteins in RBMVECs after OGD/R. **a** Immunofluorescence staining and quantitative analysis of occludin, claudin-1 and ZO-1 proteins in RBMVECs in each group. **b** Representative Western blotting images of occludin, claudin-1 and ZO-1 in RBMVECs in each group. Data are presented as the mean ± standard deviation (SD) from three independent experiments. *P < 0.001, compared with the normal group. All scale bars = 50 µm. *OGD/R* oxygen–glucose deprivation and reoxygenation. *RBMVECs* rat brain microvascular endothelial cells

The fluorescent density of occludin, claudin-1 and ZO-1 proteins in RBMVECs after OGD/R was lower than that of the normal group, and the distribution of these proteins was sparse and dispersed (Fig. 3a). Western blotting confirmed the decreased expression of these proteins in the OGD/R group compared with the normal group, and these differences were statistically significant (*P* < 0.001) (Fig. 3b). These results suggest that OGD/R treatment lowers the levels of the occludin, claudin-1 and ZO-1 proteins in RBMVECs.

### Silencing LMP2 reversed the downregulated expression of the occludin, claudin-1 and ZO-1 proteins in RBMVECs following OGD/R

**Fig. 4:**
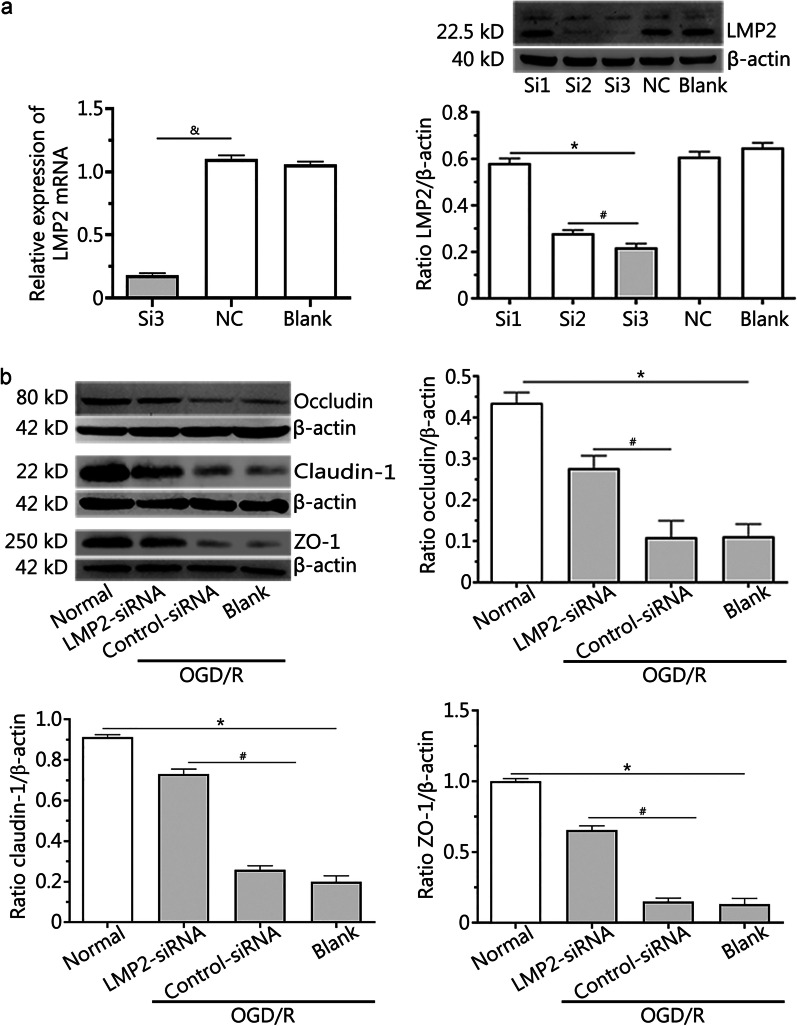
Silencing LMP2 reversed the downregulated expression of the occludin, claudin-1 and ZO-1 proteins in RBMVECs following OGD/R. **a** Transfection efficiency of LMP2-siRNA was verified by RT-PCR and Western blotting. *P < 0.001, compared with the Si1 group, ^#^*P* < 0.001, compared with the Si2 group. ^&^*P* < 0.001, compared with the NC group. **b** Western blotting showed that the levels of occludin, claudin-1 and ZO-1 proteins in RBMVECs in each group. Data are presented as the mean ± standard deviation (SD) from three independent experiments. *P < 0.001, compared with the normal group; ^#^*P* < 0.001, compared with the control-siRNA group. *OGD/R* oxygen–glucose deprivation and reoxygenation; *NC group* negative control group; *RBMVECs* rat brain microvascular endothelial cells

The results of RT-PCR and Western blotting showed that the levels of LMP2 mRNA and protein were lowest in the Si3 group (*P* < 0.001) (Fig. 4a). This indicated that Si3 was the most effective siRNA for silencing endogenous LMP2 gene expression, and its target sequence was GCACCTATATTTACGGTTA.

OGD/R induced the downregulation of occludin, claudin-1 and ZO-1 protein expression; however, this could be reversed by silencing the expression of the LMP2 gene after transfection with LMP2-siRNA. Western blotting showed that the levels of occludin, claudin-1 and ZO-1 proteins in the LMP2-siRNA group were significantly higher than those in the control-siRNA group (*P* < 0.001) (Fig. 4b).

### Silencing LMP2 rescued the downregulation of the Wnt-3a and β-catenin proteins in RBMVECs following OGD/R

**Fig. 5:**
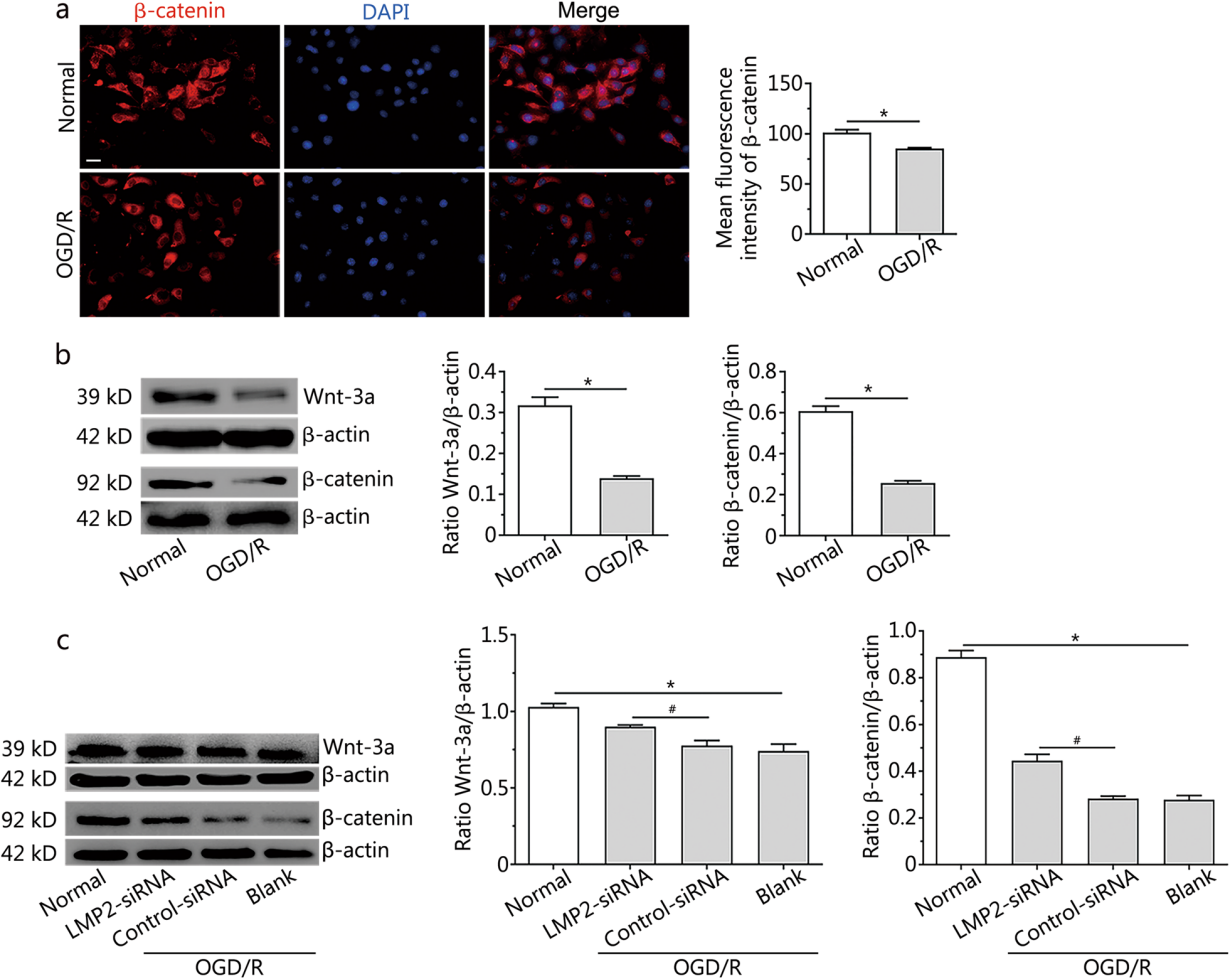
Silencing LMP2 rescued the downregulation of the Wnt-3a and β-catenin proteins in RBMVECs following OGD/R. **a** Immunofluorescence and quantitative analysis of β-catenin protein in RBMVECs under normal condition culture and OGD/R conditions. Scale bars = 50 µm. **b** and **c** Western blotting indicated that the expression of β-catenin and Wnt-3a proteins in RBMVECs in each group. Data are presented as the mean ± standard deviation (SD) from three independent experiments. *P < 0.001, compared with the normal group; ^#^*P* < 0.001, compared with the control-siRNA group. *OGD/R* oxygen–glucose deprivation and reoxygenation; *RBMVECs* rat brain microvascular endothelial cells

To verify the translocation of β-catenin between the nucleus and cytoplasm in RBMVECs during OGD/R, the expression of β-catenin was analysed by immunofluorescence staining and a Western blotting assay. The fluorescent intensity of the β-catenin protein in RBMVECs of the OGD/R group was lower than that in the control group with normal culture conditions, and the proportion of nuclear metastasis decreased (Fig. 5a). Western blotting showed that the expression of β-catenin (mean intensity value: normal group 0.61 ± 0.02 *vs.* OGD/R group 0.25 ± 0.01) and Wnt-3a (mean intensity value: normal group 0.32 ± 0.02 *vs.* OGD/R group 0.14 ± 0.01) proteins in the OGD/R group were significantly lower than that in the normal group (*P* < 0.001) (Fig. 5b); however, downregulation of these proteins could be rescued by silencing LMP2 gene expression by transfection with LMP2-siRNA. The expression levels of the Wnt-3a (mean intensity value: LMP2-siRNA group 0.90 ± 0.02 *vs.* control-siRNA group 0.77 ± 0.03) and β-catenin (mean intensity value: LMP2-siRNA group 0.45 ± 0.02 *vs.* control-siRNA group 0.28 ± 0.01) proteins in the LMP2-siRNA group were significantly higher than those in the control-siRNA group (*P* < 0.001) (Fig. 5c).

### Silencing LMP2 decreased the expression of apoptosis-related proteins and promoted the proliferation and migration of RBMVECs following OGD/R

**Fig. 6:**
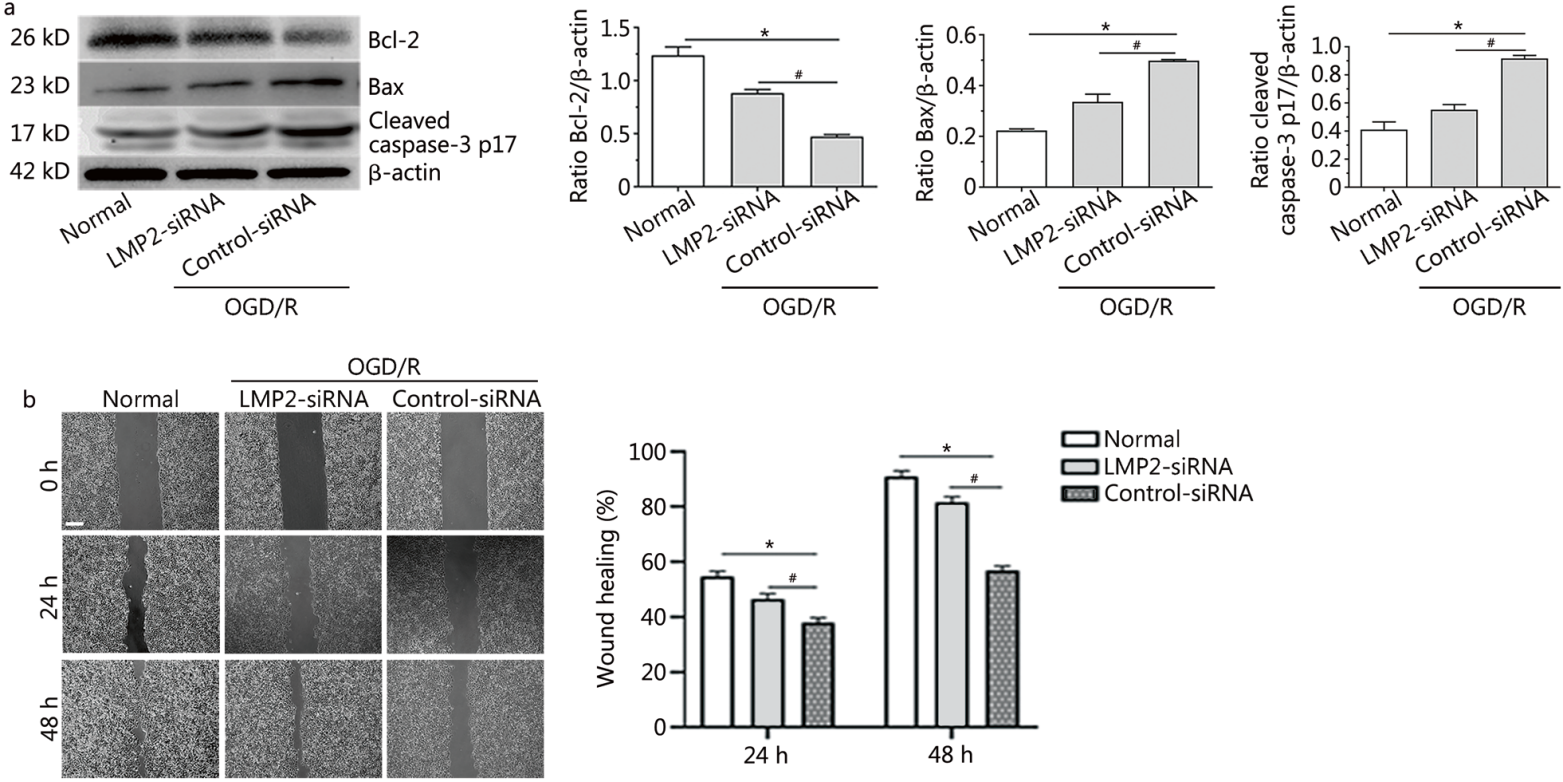
Silencing LMP2 decreased the expression of apoptosis-related proteins and promoted the proliferation and migration of RBMVECs following OGD/R. **a** Western blotting and quantitative analysis the levels of Bcl-2, Bax and cleaved caspase3-p17 proteins in RBMVECs in each group. **b** Cell scratch test showed that proliferation and migration repair ability of RBMVECs in each group. Scale bars = 250 µm. Data are presented as the mean ± standard deviation (SD) from three independent experiments. *P < 0.001, compared with the normal group; ^#^*P* < 0.001, compared with the control-siRNA group. *OGD/R* oxygen–glucose deprivation and reoxygenation; *RBMVECs* rat brain microvascular endothelial cells

We found that the expression of pro-apoptotic proteins Bax and cleaved caspase-3 p17 increased and the expression of anti-apoptotic protein Bcl-2 decreased in each group under OGD/R conditions compared with the normal culture group (*P* < 0.001). Silencing of LMP2 gene expression by transfection with LMP2-siRNA could reverse the downregulation of Bcl-2 protein and decrease the expression of Bax and cleaved caspase-3 p17 proteins compared with the control-siRNA group (*P* < 0.001) (Fig. 6a).

In the scratch migration assay, RBMVECs in the normal group showed obvious proliferation, migration and repair at 24 and 48 h after scratch injury, but this ability to migrate and repair decreased under OGD/R conditions. The cell proliferation and migration ability of RBMVECs transfected with LMP2-siRNA was increased following OGD/R compared with the control-siRNA group, and there was a significant difference in the wound healing percentage between the two groups [at 24 h, LMP2-siRNA group (57.60 ± 0.02)% *vs.* (37.90 ± 0.01)% control-siRNA group, *P* < 0.001; at 48 h, LMP2-siRNA group (81.70 ± 0.02)% *vs.* (56.90 ± 0.01)% control-siRNA group, *P* < 0.001] (Fig. 6b).

### Silencing of β-catenin aggravated downregulation of the occludin, claudin-1 and ZO-1 proteins, and reduced the proliferation and migration of RBMVECs following OGD/R

**Fig. 7:**
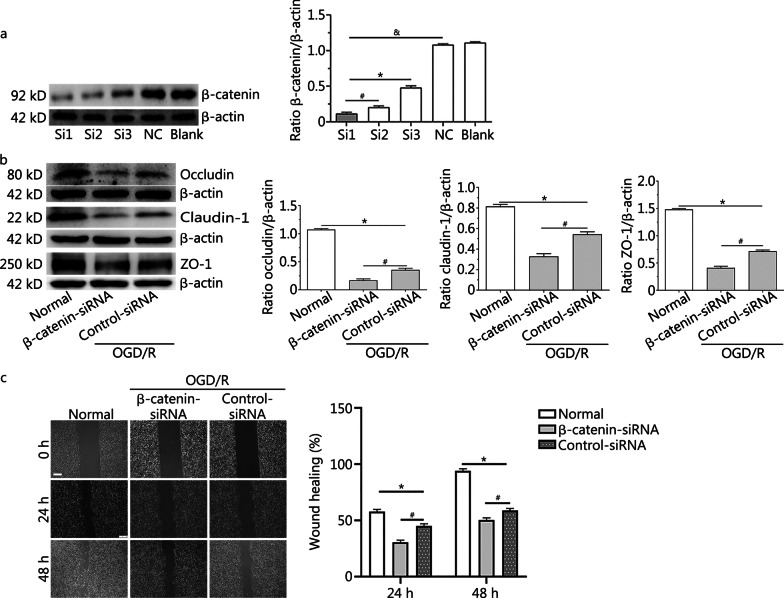
Silencing of β-catenin aggravated downregulation of the occludin, claudin-1 and ZO-1 proteins, and reduced the proliferation and migration of RBMVECs following OGD/R**. a** Western blotting showed that Si1 was the most effective siRNA for silencing endogenous β-catenin gene expression. ^&^*P* < 0.001, compared with the NC group; *P < 0.001, compared with the Si3 group; ^#^*P* < 0.001, compared with the Si2 group. **b** Representative Western blotting images of occludin, claudin-1 and ZO-1 proteins in RBMVECs in each group. **c** Cell scratch test showed that proliferation and migration repair ability of RBMVECs in each group. All scale bars = 250 µm. Data are presented as the mean ± standard deviation (SD) from three independent experiments. *P < 0.001, compared with the normal group; ^#^*P* < 0.001, compared with the control-siRNA group. *NC group* negative control group; *OGD/R* oxygen–glucose deprivation and reoxygenation; *RBMVECs* rat brain microvascular endothelial cells

Western blotting analysis showed that the expression of β-catenin protein in the Si1, Si2 and Si3 groups was significantly lower than that in the negative control group, with the expression of β-catenin protein being lowest in the Si1 group (*P* < 0.001) (Fig. 7a). This suggested that the most effective sequence for inhibiting the expression of endogenous β-catenin was Si1, with a target sequence of: ACATCGAAGACTCTACAAT.

Expression of the occludin, claudin-1 and ZO-1 proteins in cells under OGD/R conditions was significantly decreased compared with the control group under normal conditions. Silencing of β-catenin gene expression by transfection with β-catenin-siRNA aggravated the downregulation of these proteins. The expression levels of the occludin, claudin-1 and ZO-1 proteins in the β-catenin-siRNA group were significantly lower than in the control-siRNA group (*P* < 0.001) (Fig. 7b).

In the scratch migration assay under normal conditions, RBMVECs showed obvious proliferation, migration and reparation at 24 and 48 h after ‘scratch injury’, and the wound healing percentage was (57.60 ± 0.02)% and (93.90 ± 0.02)% at 24 and 48 h, respectively. However, OGD/R inhibited the migration of RBMVECs. Furthermore, compared with the control-siRNA group [with wound healing percentages of (37.90 ± 0.02)% and (58.80 ± 0.01)% at 24 and 48 h, respectively], the proliferation and migration ability of RBMVECs transfected with β-catenin-siRNA was significantly decreased, showing wound healing percentages of (30.40 ± 0.01)% and (50.10 ± 0.02)% at 24 and 48 h, respectively (*P* < 0.001) (Fig. 7c). This suggested that silencing of the β-catenin gene further inhibited the migration of endothelial cells under OGD/R conditions.

### Effects of combined silencing of the β-catenin and LMP2 genes on the expression of occludin, claudin-1 and ZO-1 proteins in RBMVECs following OGD/R

**Fig. 8:**
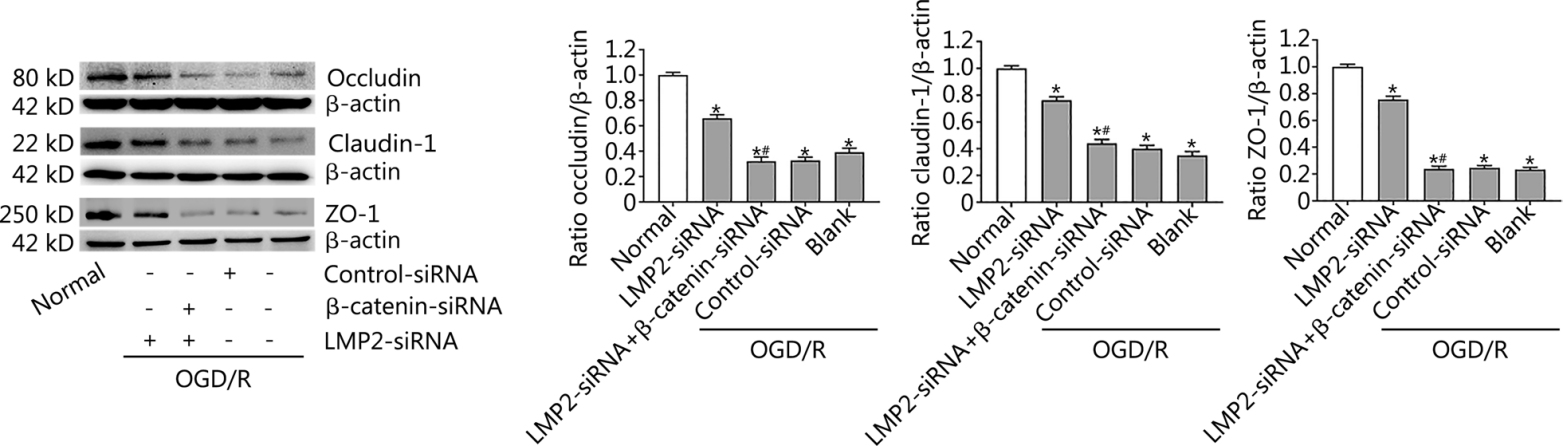
Effects of combined silencing of the β-catenin and LMP2 genes on the expression of occludin, claudin-1 and ZO-1 proteins in RBMVECs following OGD/R**.** Western blotting and quantitative analysis of the expressions of occludin, claudin-1and ZO-1proteins in RBMVECs in each group. Data are presented as the mean ± standard deviation (SD) from three independent experiments. *P < 0.001, compared with the normal group; ^#^*P* < 0.001, compared with the LMP2-siRNA group. *OGD/R* oxygen–glucose deprivation and reoxygenation. *RBMVECs* rat brain microvascular endothelial cells

To further understand whether the immunoproteasome affects the expression of BBB-related proteins by regulating the Wnt/β-catenin signal pathway, LMP2-siRNA and β-catenin-siRNA were co-transfected with RBMVECs to observe the expression of occludin, claudin-1 and ZO-1 proteins in RBMVECs under OGD/R conditions. We found that the downregulation of occludin, claudin-1 and ZO-1 proteins in RBMVECs exposed to OGD/R could be reversed by transfection with LMP2-siRNA, but this beneficial effect was partly counteracted by co-transfection with β-catenin-siRNA (*P* < 0.001) (Fig. 8).

## Discussion

BBB dysfunction is a prominent pathological characteristic of acute ischemic stroke, thereby making it a potentially promising therapeutic target in ischemic stroke [2]. The molecular mechanisms of BBB damage after a stroke include modification of the expression of tight junctions and membrane transporter proteins, and the inflammatory response [3]. Injury to the BBB contributes to worsening stroke neurological outcomes and poor prognoses. Therefore, it is vital to protect against ischemia-induced BBB injury. Although the neuroprotective efficacies of many agents have been confirmed in animal stroke models during the last few decades, none have been successfully applied clinically. Recent research suggests that therapeutic agents based on interference with multimodal cell cascades are superior to those targeting a single specific pathway in experimental stroke therapy [1, 2]. Thus, the ubiquitin–proteasome system is regarded as an ideal candidate target for drug therapy, as it is involved in multiple cellular pathways. The proteasome is a multicatalytic protease complex that is critically involved in various physiological and pathological processes, including the cell cycle, apoptosis, cell signalling and inflammatory reactions [19]. It has previously been reported that proteasome inhibition displays neuroprotection against cerebral ischemia in rodent models [20]. Proteasome inhibitors, such as MLN519, VELCADE and BSc2118, successfully inhibit inflammation and contribute to maintaining BBB integrity after haemorrhagic or ischemic stroke [6, 7]. However, the effects of proteasome inhibition are two-sided. Proteasome malfunction after cerebral ischemia leads to the aggregation of misfolded proteins and ultimately cell death [21]. The clinical application of general proteasome inhibitors is often limited by their side effects due to their nonselective inhibition of protein degradation.

By contrast to the standard proteasome, which is constitutively expressed in the brain, the immunoproteasome is generally expressed at low levels under non-stimulating conditions, but can be induced when cells are exposed to various factors such as ischemia, oxidative stress [22] and inflammation [23]. Immunoproteasome-mediated proteolysis has emerged as an important molecular mechanism for regulating wide-ranging functions, including immune and non-immune functions [22]. Excessive and prolonged induction of immunoproteasome activity can be detrimental to tissue and has been associated with the pathogenesis of autoimmune disease [24], hypertension [25] and cerebral ischemia [26]. Our previous work observed that inhibition of immunoproteasome LMP2 significantly reduced the cerebral infarction volume, attenuated inflammatory reactions, enhanced angiogenesis and improved neurological function recovery in rat stroke models [9, 10]. In addition, we found higher levels of plasma immunoproteasome in ischemic stroke patients with haemorrhagic transformation [13, 14]. Increased BBB permeability is the main feature of cerebral infarction haemorrhage transformation. Therefore, we postulated that the immunoproteasome might, through a series of complex mechanisms, affect BBB integrity and secondary haemorrhagic transformation of the ischemic area. The overall increase in BBB permeability after a stroke is closely linked to the decreased expression of tight junction proteins, such as claudin-5, occludin and ZO-1, among others. In this study, we found that inhibition of immunoproteasome LMP2 enhanced the levels of ischemia-induced components of the BBB, upregulating occludin, claudin-1 and ZO-1 protein expression, improving BBB permeability, and promoting the upregulation of Wnt-3a and β-catenin proteins in rats after MCAO/R. In vitro experiments revealed that oxygen–glucose deprivation/reoxygenation caused the upregulation of LMP2 and downregulation of the occludin, claudin-1 and ZO-1 proteins in RBMVECs. However, these changes were reversed after silencing LMP2 gene expression by transfection of RBMVECs with LMP2-siRNA. After silencing LMP2 gene expression, both the levels of occludin, claudin-1 and ZO-1 proteins and the proliferation and migration ability of RBMVECs were significantly improved. These results suggest that the immunoproteasome modulates the degradation of these proteins. Together, these data highlight the important role of LMP2 inhibition in maintaining the integrity of BBB under ischemic conditions.

The unique microenvironment of the CNS is tightly regulated by the integrity of the BBB. However, the molecular mechanisms that regulate CNS angiogenesis and BBB formation are largely unknown. Canonical Wnt/β-catenin signalling during embryonic and postnatal development is fundamental in mediating brain development, CNS vascularization, BBB formation and maturation [27–29]. During the activation of Wnt/β-catenin signalling, the expression of various Wnt ligands (Wnt-1, Wnt-3a, Wnt-5a) and β-catenin proteins increase. Endothelial β-catenin signalling is required for maintenance of the BBB integrity and CNS homeostasis [30]. β-catenin is a component of endothelial tight junctions, and its loss may reduce junction stability and increase vascular permeability. It was observed that claudin-1 and claudin-3 were downregulated in the brain endothelial cells of mice that also possessed conditional knockout of the β-catenin gene, which indicated that β-catenin transcriptionally controls the expression of tight junction proteins claudin-1 and claudin-3 [31]. Interestingly, endothelial Wnt/β-catenin signalling reduced immune cell infiltration and partially restored functional BBB integrity in multiple sclerosis [32]. In addition, it was found that oxygen–glucose deprivation led to apoptosis of brain microvascular endothelial cells accompanied by a decrease in the expression of Wnt-3a mRNA [33]. Similarly, the present study found that OGD/R caused the downregulation of Wnt-3a and β-catenin proteins accompanied by a reduction of occludin, claudin-1 and ZO-1 protein expression, which indicated that Wnt/β-catenin signalling is necessary for the production of BBB structural proteins occludin, claudin-1 and ZO-1. This phenomenon could be further validated after silencing of β-catenin gene expression by transfection with β-catenin-siRNA. In addition, we found that the proliferation and migration ability of RBMVECs transfected with β-catenin-siRNA was significantly decreased compared with the negative control group, suggesting that silencing of the β-catenin gene further inhibited the migration of endothelial cells under the condition of OGD/R. It was recently reported that upregulation of Wnt/β-catenin components was accompanied by vascular repair after traumatic brain injury [34]. Therefore, it may be concluded that the Wnt/β-catenin signal is necessary for BBB formation, maturation and reparation after ischemia-induced brain injury.

It is worth noting that the ubiquitin–proteasome system controls the activation of Wnt/β-catenin signalling. Without Wnt stimulation, cytoplasmic β-catenin levels remain low. Phosphorylation of β-catenin mediated by glycogen synthase kinase-3β (GSK3β) allows for binding of the E3 ubiquitin ligase β-TrCp, which finally marks the protein for proteasomal degradation. The immunoproteasome is a subtype of proteasome that possesses broader biological functions. It regulates proinflammatory cytokine production, and T cell differentiation and proliferation. Alongside immune functions, the immunoproteasome has been demonstrated to efficiently clear proteins by its enhanced proteolytic activity in both a ubiquitin-dependent and -independent manner. Inhibition of immunoproteasome LMP2 promotes angiogenesis and enhances HIF-1α accumulation in a rat model of cerebral ischemia [10], supporting the conclusion that the immunoproteasome possesses proteolytic activity involved in protein degradation. Several studies reported negative regulation of Wnt/β-catenin by nuclear factor kappa-B (NF-κB) [35]. NF-κB activation can inhibit β-catenin/TCF activity through upregulation of leucine zipper tumour suppressor-2 or promotion of β-catenin degradation through induction of the E3 ubiquitin protein ligases SMAD ubiquitination regulatory factor-1 (Smurf1) and Smurf-2. Interestingly, inhibition of immunoproteasome LMP2 can also decrease the expression of NF-κB under stroke conditions [9]. Therefore, it is possible that the immunoproteasome also indirectly inhibits Wnt/β-catenin signalling through the activation of NF-κB.

The present study has some limitations. For example, the immunoproteasome has three specific catalytic subunits with apparent diverse functions. The present study focused on LMP2 and investigated whether inhibition of immunoproteasome LMP2 protects the BBB and the mechanisms involved in this neuroprotective effect. Previous studies have reported that LMP7 plays an important role in some disease models [36, 37]. In addition, the duration of cerebral ischemia/reperfusion was two weeks in our study. Angiogenesis after cerebral ischemia is a lengthy process. Undoubtedly, a longer duration of cerebral ischemia/reperfusion would be favourable. Further studies are required to fully understand the mechanism by which the immunoproteasome exacerbates ischemic injury.

## Conclusions

The present study demonstrates that inhibition of immunoproteasome LMP2 effectively reversed ischemia/hypoxia-induced downregulation of tight junction proteins such as occludin, claudin-1 and ZO-1, and improved BBB integrity. The molecular mechanism may involve the immunoproteasome-regulated activation of the Wnt/β-catenin signalling pathway under ischemic conditions.

## Supplementary Information

**Additional file 1: Fig. S1**. Rat brain microvascular endothelial cells (RBMVECs) were cultured and identified. **a** Rat brain microvascular endothelial cells (RBMVECs) were observed under the inverted microscope (Scale bars, left 250 µm, right 50 µm). **b** RBMVECs were confirmed with immunofluorescence of vascular von Willebrand factor (VWF). Scale bars = 50 µm.

**Additional file 2: Fig. S2**. Immunofluorescence staining showed the expressions of LMP2 and CD31 in rat brain cortex tissue. Some LMP2 positive cells were colocalized with CD31-positive vascular endothelial cell (arrow). Scale bars = 50 µm.

## Data Availability

The datasets used and/or analysed during the current study are available from the corresponding author on reasonable request.

## Abbreviations

BBB: Blood–brain barrier
CNS: Central nervous system
DMEM: Dulbecco’s modified eagle medium
GSK3β: Glycogen synthase kinase-3β
LMP2: Low molecular mass peptide 2
MCAO/R: Middle cerebral artery occlusion/reperfusion (MCAO/R)
NF-κB: Nuclear factor kappa-B
OGD/R: Oxygen–glucose deprivation/reperfusion
RBMVECs: Rat brain microvascular endothelial cells
RT-PCR: Reverse transcription-polymerase chain reaction
SDS/PAGE: Sodium dodecyl sulphate / polyacrylamide gel electrophoresis
shRNA: Short hairpin RNA
Smurf: SMAD ubiquitination regulatory factor
UPS: Ubiquitin–proteasome system
VWF: Vascular von willebrand factor
ZO: Zonula occluden
