# Supplementary figures and images for: Inhibition of the immunoproteasome LMP2 ameliorates ischemia/hypoxia-induced blood–brain barrier injury through the Wnt/β-catenin signalling pathway
Source: Mil Med Res. 2021 Dec 3;8:62. doi: 10.1186/s40779-021-00356-x (PMC8641178; doi:10.1186/s40779-021-00356-x)

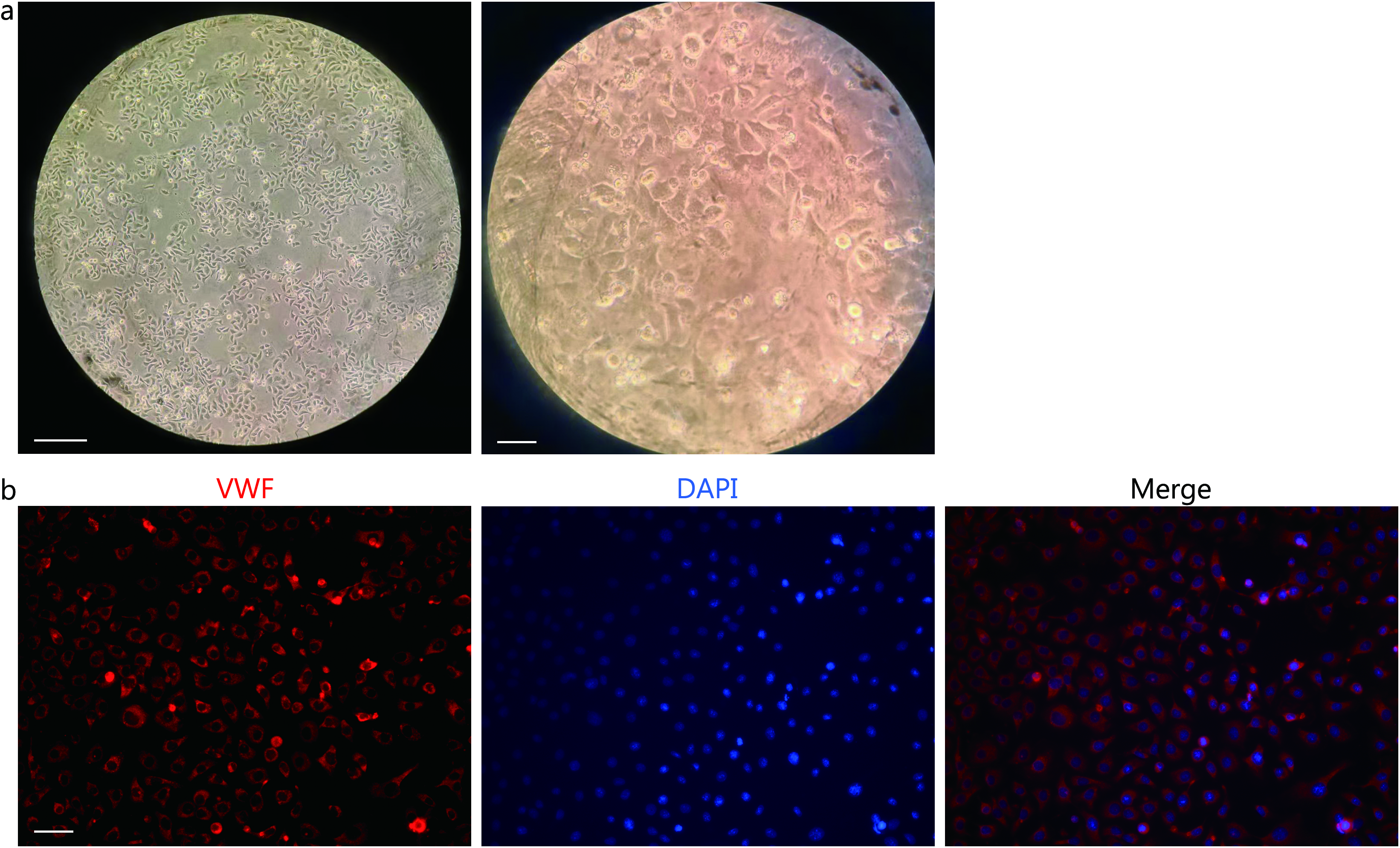

Supplement: Supplementary file 1 — Additional file 1: Fig. S1. Rat brain microvascular endothelial cells (RBMVECs) were cultured and identified. a Rat brain microvascular endothelial cells (RBMVECs) were observed under the inverted microscope (Scale bars, left 250 µm, right 50 µm). b RBMVECs were confirmed with immunofluorescence of vascular von Willebrand factor (VWF). Scale bars = 50 µm. [file 40779_2021_356_MOESM1_ESM.tif]

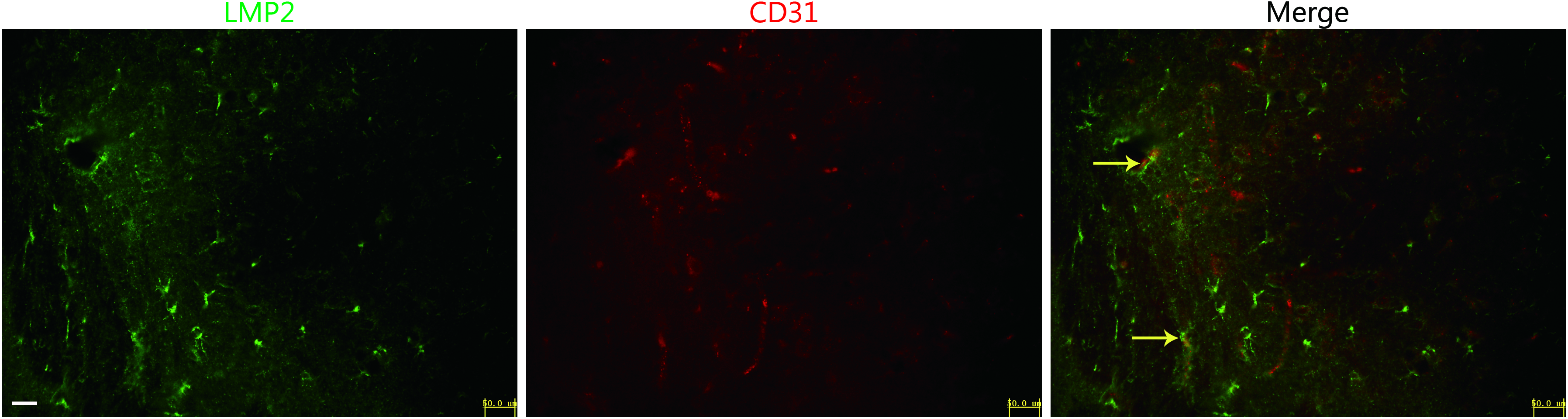

Supplement: Supplementary file 2 — Additional file 2: Fig. S2. Immunofluorescence staining showed the expressions of LMP2 and CD31 in rat brain cortex tissue. Some LMP2 positive cells were colocalized with CD31-positive vascular endothelial cell (arrow). Scale bars = 50 µm. [file 40779_2021_356_MOESM2_ESM.tif]
